# RNAMotifComp: a comprehensive method to analyze and identify structurally similar RNA motif families

**DOI:** 10.1093/bioinformatics/btad223

**Published:** 2023-06-30

**Authors:** Md Mahfuzur Rahaman, Nabila Shahnaz Khan, Shaojie Zhang

**Affiliations:** Department of Computer Science, University of Central Florida, Orlando, FL 32816, United States; Department of Computer Science, University of Central Florida, Orlando, FL 32816, United States; Department of Computer Science, University of Central Florida, Orlando, FL 32816, United States

## Abstract

**Motivation:**

The 3D structures of RNA play a critical role in understanding their functionalities. There exist several computational methods to study RNA 3D structures by identifying structural motifs and categorizing them into several motif families based on their structures. Although the number of such motif families is not limited, a few of them are well-studied. Out of these structural motif families, there exist several families that are visually similar or very close in structure, even with different base interactions. Alternatively, some motif families share a set of base interactions but maintain variation in their 3D formations. These similarities among different motif families, if known, can provide a better insight into the RNA 3D structural motifs as well as their characteristic functions in cell biology.

**Results:**

In this work, we proposed a method, RNAMotifComp, that analyzes the instances of well-known structural motif families and establishes a relational graph among them. We also have designed a method to visualize the relational graph where the families are shown as nodes and their similarity information is represented as edges. We validated our discovered correlations of the motif families using RNAMotifContrast. Additionally, we used a basic Naïve Bayes classifier to show the importance of RNAMotifComp. The relational analysis explains the functional analogies of divergent motif families and illustrates the situations where the motifs of disparate families are predicted to be of the same family.

**Availability and implementation:**

Source code publicly available at https://github.com/ucfcbb/RNAMotifFamilySimilarity.

## 1 Introduction

The availability of 3D structural data for different types of RNAs is increasing gradually. The understanding of these 3D structural data uncovers a new dimension to RNA-based research and therapeutics (Li et al. 2020). Due to their participation in various biological functions (Eddy 2001; Storz 2002; Wan et al. 2011; Rinn and Chang 2012; Wang et al. 2018), ncRNAs are becoming one of the major focuses of RNA research in recent days. Although the 3D structure of an RNA is formed by nucleotide building blocks, their biological functions are mostly defined by their recurrent structural segments which are referred to as the building blocks of RNA structure (Moore 1999; Hendrix et al. 2005; Leontis et al. 2006). These recurrent structural segments are designated as RNA structural motifs. The structural motifs could be located in both helical regions and loop regions of an RNA. For this work, we are focusing on the structural motifs from the loop regions only. Based on the structural similarities, the structural motifs from the loop regions are divided into several motif families. A few of these families are well-studied and there are still a significant number of motif families that are not even named yet.

**Figure 1.**
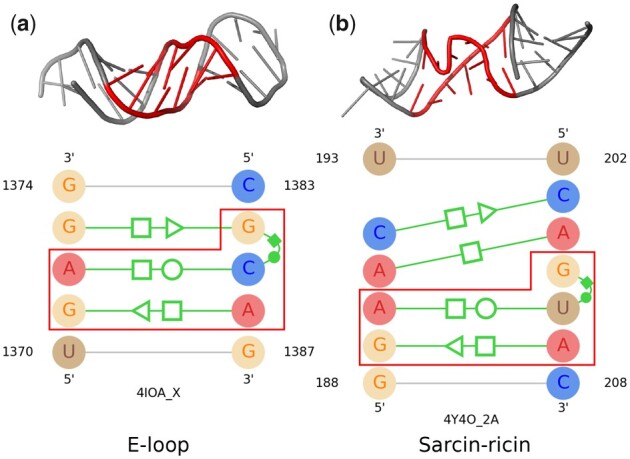
The 3D structure and base-pair annotation of (a) an E-loop motif instance, and (b) a Sarcin-ricin motif instance. Similar interactions between the two instances are highlighted using red boxes in both images.

RNAMotifScan (Zhong et al. 2010), RNAMotifScanX (Zhong and Zhang 2015), and FR3D (Sarver et al. 2008) analyze the 3D structures of the non-redundant RNA chains to find the instances of well-known motif families. Zhong and Zhang (2012) and Ge et al. (2018) found recurrent structural motifs by clustering motif instances based on RNAMotifScan (Zhong et al. 2010). Petrov et al. (2013) cluster similar structures by geometrically comparing them using the FR3D (Sarver et al. 2008) tool. In most cases, these structures in a cluster or group are almost the same in shape and are constructed with the same isosteric base interactions. But there also exists variation in the structure of the instances from the same motif family. RNAMotifContrast (Islam et al. 2021) focused on these variations and introduced the concept of subfamilies. The instances of the same motif family can be subdivided into different subfamilies based on the variations in their 3D structures. On the other hand, similar isosteric base interactions can form different 3D structures as well. As a result, different motif families might share the same arrangement of base interactions. One such example is shown in Fig. 1. In this figure, the first motif instance is taken from the E-loop and the other one is taken from the Sarcin-ricin motif family. As the figure demonstrates, even though the two motifs share three base-pairing interactions, they form different 3D structures in RNA. This type of similarity in different motif families and variation among the same motif family adds extra complexity to the computational identification process of RNA 3D structural motifs.

In this work, we have proposed a method, RNAMotifComp, that analyzes the similarities in 3D structures among the input instances of different structural motif families of RNAs and generates a similarity graph to represent the relation among the known families. This similarity is analyzed not only based on their 3D formations but also from their isosteric base-interaction point of view. RNAMotifComp takes two or more motif families as input and generates a similarity graph by analyzing the input motif instances to show if they have any structural resemblance. It also identifies the instances that are similar to the instances of another motif family and writes them in separate text files as output for further analysis. To find the similarities from the interaction point of view, we employed the alignment tool from RNAMotifScanX (Zhong and Zhang 2015) that actually uses a graph alignment algorithm based on the base interactions. It considers the non-canonical interactions and also the sequence conservation of the motifs. Alternatively, for shape-based similarity, we have utilized the RNA-align (Gong et al. 2019) tool, which works based on a length-independent scoring function TM-score (Zhang and Skolnick 2004). TM-score utilizes 3D coordinates of the structures to measure the similarity between two instances and so, this similarity type is referred to as coordinate-based similarity throughout the manuscript. From the outcome of our analysis, we have summarized the resemblance of the known motif families and provided benchmarking data to our claim by using RNAMotifContrast on an unannotated set of structural motif instances. We have also shown the effect of this structural similarity on motif identification using a machine-learning model on a selected set of known motif families.

## 2 Materials and methods

To show the similarities among different motif families using RNAMotifComp, we have utilized a dataset of known RNA structural motif families. The same motif instances were used to benchmark our analysis. To find the 3D structural resemblance from the base interaction point of view, we have employed the alignment tool from RNAMotifScanX. On the other hand, to explore the similarities from the coordinate point of view, we have utilized the RNA-align alignment tool. In both cases, the alignment results are used to calculate the Root-Mean-Square Deviation (RMSD) values between two motif instances. The closeness of any two motifs is determined by considering the alignment result (both RMSD and alignment length from interaction or coordinate-based alignment). RNAMotifContrast is used as a clustering tool on an unannotated motif dataset to verify the results produced from RNAMotifComp. We have also applied a basic Naïve Bayes classifier to show the effect of structural similarity in motif family identification. A brief description of the dataset, procedures of RNAMotifComp, and the benchmarking steps are given in the following subsections.

### 2.1 Collect known motif family instances

We have used a total of 360 instances of internal loop motifs from 11 well-known structural motif families for this work. The distribution of these motif instances is given in Table 1. From these instances, nine motifs have HETATM entries in the PDB file of the corresponding motif region. As we were facing difficulties working with HETATMs when generating the alignments, we excluded these instances from our analysis. Additionally, we have filtered the potential outlier motifs by calculating their z-score based on the respective alignment results of all motif instances. In case of interaction based alignment, seven such instances were filtered out based on their low z-score while the number became six in the case of coordinate-based alignment. The low z-score represents poorly aligned instances with other members of the same family and they are identified as structurally very different. The main source of these input motif instances is the De Novo clustering work of Ge et al. (2018) and the RNA 3D Motif Atlas database (Petrov et al. 2013). The curation process explained in the RNAMotifContrast paper (Islam et al. 2021) is followed to get detailed information on the internal loop motif instances from this dataset. The locations of these motif instances are collected from RNAMotifContrast as well and provided in our source repository as “IL.in” under the “data” directory. As our dataset is focused on internal loops only, any hairpin stem-loops associated with the input motif instances are not considered as part of the motif family.

**Table 1.** The distribution of motif instances of well-known motif families used for RNAMotifComp.

Motif family	Number of motif instances
Kink-turn (KT)	67
reverse Kink-turn (rKT)	8
Sarcin-ricin (SR)	74
C-loop (CL)	44
E-loop (EL)	49
Hook-turn (HT)	34
Tandem-shear (TS)	45
Tetraloop-receptor (TR)	19
L1-complex (L1C)	6
Rope-sling (RS)	12
T-loop (TL)	2

### 2.2 Annotate motifs and extract coordinates

To generate the interaction-based alignment between two motifs using RNAMotifScanX, we needed to provide the base-pairing and base-stacking interactions along with their nucleotide sequences. To do so, we have downloaded all the PDB files corresponding to the motif locations that were used as input for our analysis. We then generated the annotations for all these PDBs using the DSSR (Lu et al. 2015) annotation tool and also downloaded the corresponding FR3D (Sarver et al. 2008) annotation files. We have merged the two annotation results for each PDB file to get more precise annotations of base interactions. Due to the merging of two different annotation data, we found some conflicts in the annotation. For example, two annotation tools are predicting different base pairs in the same locations or the same edge of a single nucleotide interacting with edges from two different nucleotides. We resolved these conflicts by taking the interactions that appeared more often than the other throughout the base interactions of each RNA chain from the annotations of all PDBs in the non-redundant PDB list release 3.57 at resolution 4.0 Å. Based on the base-pairing and base-stacking annotation data, we have generated separate files for each motif instance with their interaction and sequence data. These files were used as input to produce alignment results using RNAMotifScanX.

RNA-align aligns the motif instances based on the TM-score and it uses the 3D coordinates of the backbone atom while doing the alignments. Moreover, it requires the whole PDB files to work with and RNA-align aligns the first chains of each PDB file. To simplify the process and to generate the alignments for specific motif locations only, we have to extract those locations and generate partial PDB files for each motif instance. These partial PDB files were utilized by RNA-align to generate the coordinate-based structural alignments.

### 2.3 Calculate RMSD of two motif instances

While doing the structural alignment of two motif instances, we get an aligned set of nucleotides as the alignment result. Alignment length is the total number of aligned nucleotides of that outcome excluding any insertion and deletions. In both types of alignment cases (interaction-based and coordinate-based), we have considered the coordinates of all heavy atoms from the backbone and the sugar ribose for the aligned nucleotides. We have calculated the centroid of these heavy atoms and then calculated the RMSD from the centroid to centroid using the following formula: where *N* is the total number of atoms, and $\delta_{i}$ is the distance between corresponding aligned atoms at position *i*.


$$\mathrm{RMSD}=\sqrt{\frac{1}{N}\sum_{i=1}^{N}\delta_{i}^{2}}$$

We have used two different alignment tools to show the relationships among the structural motif families from two different points of view. The alignment results are then utilized to calculate the RMSD between two motifs. A key point to note here is that the RMSD value is not length independent and the value can vary based on the alignment length. To handle this scenario and to make sure the two motifs with low RMSD are actually structurally similar, we considered the alignment length threshold for both motif families while measuring the similarity. We made sure the alignment length is equal to or higher than the threshold of both motif families so that their own structural features could be conserved. Another key concern of using two different alignment tools is that the tools are not performing the same type of alignment. RNAMotifScanX aligns the interactions and provides locally aligned regions as output. On the other hand, RNA-align aligns the structure using their 3D coordinates and their result is a global alignment. As the working criteria for the two alignment tools are quite different, it is highly likely to have a bit of divergence in the alignment result as well as in the RMSD value for a specific pair of motif instances. In both cases, RMSD is calculated based on the aligned nucleotides only. As RNAMotifScanX performs local alignment and RNA-align performs global alignment, the RMSD value found from the RNA-align alignment result would be comparatively higher than the RMSD value calculated from the RNAMotifScanX alignment. We have considered this imparity while setting the cutoff value for RNAMotifComp.

### 2.4 Compute similarities between two motif families

In RNAMotifComp, two different motif families are defined as similar if a subset of the instances of one motif family is found structurally closer to a subset of another motif family. The closeness is measured from two different points of view: interaction-based and coordinate-based. The two participating subsets from two different motif families closely aligned in the interaction based alignment result with a lower average RMSD value based on their aligned region are identified as structurally similar from interaction point of view. Similarly, the two participating subsets will be similar from the coordinate point of view if they are better aligned to each other while using coordinate based alignment tool and also they have low average RMSD value. The process of calculating RMSD for both interaction-based and coordinate-based alignment is discussed in the previous subsection. At the beginning of our analysis, we generated all pair alignments between two well-known motif families. To focus on the interaction-based structurally similar instances, after generating all pair alignments between two motif families, we selected only the best-aligned pairs based on the following criteria. The first step is to select the alignments for which the alignment length is greater or equal to both family-specific alignment length thresholds. This family-specific alignment length threshold is calculated by taking the average of all pair alignments among the instances of the same motif family. The family-specific threshold implies that, if a motif instance is to be considered a member of a specific motif family, it needs to have an alignment length of equal or higher value compared to the threshold value of that particular family. The next step for an alignment pair to be considered as best alignment is having an RMSD value greater than or equal to a specific threshold. From our experimental data based on the RNAMotifScanX alignment, on average around 60% of all intra-family motif pairs have RMSD 1.0 Å or better. The percentage is slightly lower for Kink-turn, reverse Kink-turn, C-loop, E-loop, and Hook-turn motif families due to the high internal variation that can be confirmed by the subfamiliy distribution from RNAMotifContrast (Islam et al. 2021) paper. Besides, on average, only around 15% of the inter-family motif pairs have RMSD ≤ 1.0 Å while 85% of them have RMSD > 1.0 Å. This indicates, the inter-family motif pairs tend to have RMSD > 1.0 Å in case of interaction-based alignment and increasing the threshold value will show more inter-family pairs as better alignment. So, we choose RMSD 1.0 Å as our threshold value along with the family-specific alignment length threshold to consider a motif of another family as similar to the one from the reference family using interaction-based alignment. For the coordinate-based alignment, the same steps are followed as described above except for the fact that we increased the RMSD threshold to 1.5 Å as RNA-align does global alignment unlike the local alignment done by RNAMotifScanX. Due to the global alignment, some nucleotides might not be well aligned and affect the RMSD to be a bit higher than the locally aligned version. Our experimental data also shows that for coordinated-based alignment, 60% intra-family motif pairs are considered better aligned using 1.5 Å RMSD threshold, while 24% of the inter-family pairs have RMSD < 1.5 Å on average. The RMSD distribution for all intra-family and inter-family motif pairs from our experimental data using both interaction-based and coordinate-based alignment is given in Supplementary Tables S1 and S2.

**Figure 2.**
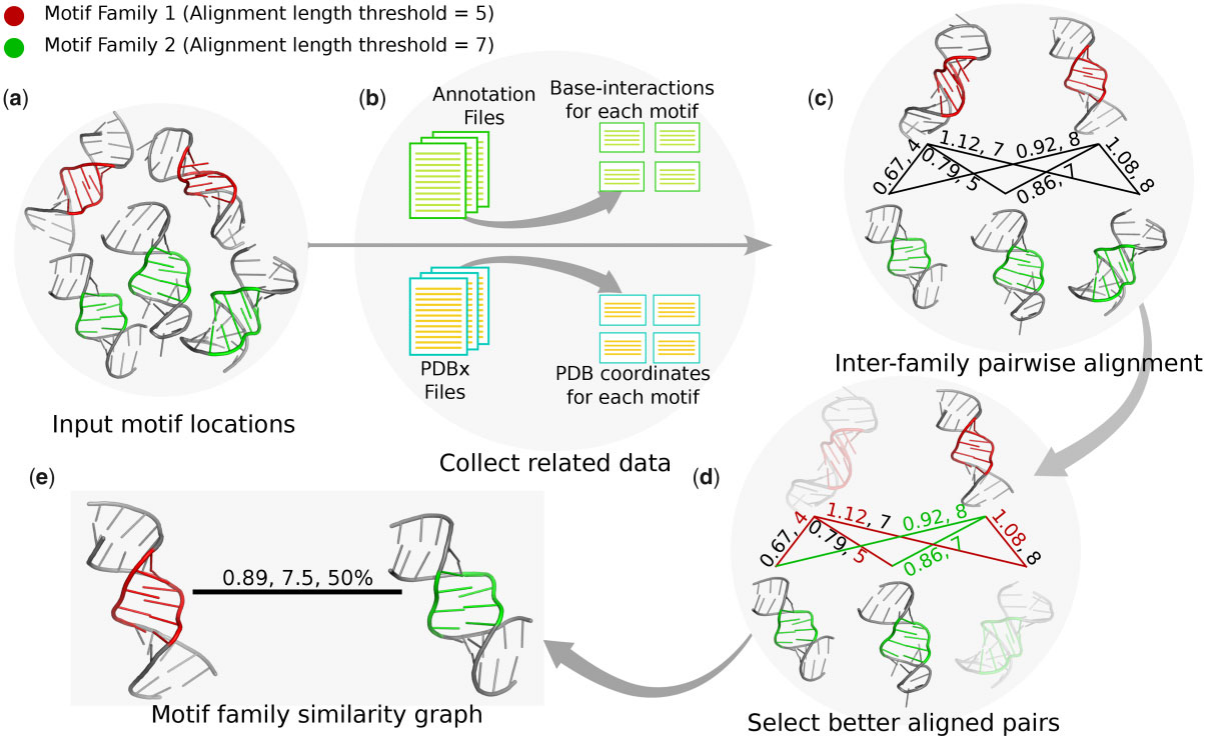
A simplified representation of RNAMotifComp pipeline. Red and green colored motifs represent the instances from two different motif families. (a) Input motif instances, (b) visual representation of utilizing PDBx files and corresponding annotation files to get the coordinates and base-interactions of each motif, respectively, (c) generate inter-family pair-wise alignments to identify similar structures, (d) select the participating instances from each family, (e) a visual representation of the similarity graph; here, each family is represented with a single motif.

Now, for these best-aligned pairs between two families, we identified the number of instances for each family that is taking part in the alignments as these are similar to instances from another motif family. We calculated the percentage of these participating instances for each motif family. We are saying two motif families are similar if the percentage of participating instances from each family is 20% or higher (for both interaction-based and coordinate-based alignment). In the similarity graph that we have prepared, the minimum of these two percentage values is added as the edge label along with the average RMSD and average alignment length of the selected alignment pairs. Figure 2 graphically shows the whole procedure of RNAMotifComp with a simplified example of two motif families where the RMSD threshold is set to 1.0 Å. While using RNAMotifComp, an user will have the option to adjust both RMSD and percentage of participating instances threshold values to get more or less strict similarities among their input motif families.

### 2.5 Benchmark using RNAMotifContrast

We have utilized RNAMotifContrast to group similar motif instances from an unannotated dataset. Here, by unannotated we are referring to the motif instances without their family information. To get the unannotated version, we merged all the instances of different motif families and provided them as a single family to RNAMotifContrast. RNAMotifContrast normally takes a single family as input and subdivides the motifs of that family into separate groups which are then defined as subfamilies. The motif instances of a subfamily have higher similarity than the instances of the other one. We have utilized this feature of clustering similar motif instances from RNAMotifContrast to group the similar motifs from our unannotated input instances. We have generated the clusters using both interaction-based and coordinate-based alignment. There are several parameters to control the output of RNAMotifContrast, and the “connectivity test threshold” is one of them. This parameter is used to control the merging criteria of Connected Motif Groups (CMG) in RNAMotifContrast. If the value is higher, it will be less likely to merge multiple CMGs into one and vice versa. As we wanted to decrease the rate of merging the subclusters, we changed the connectivity test threshold from 50% to 80% for both cases (interaction-based and coordinate-based alignment). The “RMSD threshold for merging” is another parameter that controls whether to merge two CMGs based on a cutoff value, the default is set to 1.0 Å in RNAMotifContrast. To match the cutoff with our experimental RMSD threshold (which is discussed in the “Compute similarities between two motif families” subsection), we kept the default value of this parameter for interaction-based benchmarking and used the cutoff of 1.5 Å for coordinate-based benchmarking. All other parameter values were kept the same as the default ones. The outcome of RNAMotifContrast is then annotated using the family information from our original input dataset.

### 2.6 Evaluate utility using Naïve Bayes classifier

As a representative of various machine learning models, we have used a basic classifier to show the utilities of RNAMotifComp. Due to the independent nature of features and supposedly Gaussian distribution of continuous feature values, the Gaussian Naïve Bayes (GNB) Classifier is selected. A variance smoothing of 1e-9 is used to improve the accuracy of the classifier. As our main objective is to demonstrate the consequences of having similar motif instances across motif families using this GNB classifier, and since it is important to have a good amount of instances in each class, we handpicked some particular motif families to train our machine-learning model. Here, for two different types of alignments (interaction-based and coordinate-based), we have performed two different types of analysis, referred to as Interaction-based GNB (IGNB) analysis and Coordinate-based GNB (CGNB) analysis. A summary of the similar motif families identified by RNAMotifComp is shown in Table 2 under Results section. From this table, we have selected similar motif families based on interaction-based alignment and a single motif family that is non-similar to them to show the effect of similarity in our IGNB analysis. These similar motif families include EL, TS, and KT while the selected non-similar one is SR. SR is selected over the other non-similar motif families since it has comparatively the highest number of motif instances among them. Similarly, for the CGNB analysis, EL, TS, HT, CL, and TR are selected as similar motif families and SR is again selected as a non-similar motif family. Later, we excluded TR from this set of similar families due to having a low instance count which might not be enough to train a machine-learning model.

**Table 2.** Similar motif family pairs identified by RNAMotifComp.

3D structural similarity criteria	Motif family pairs
Interaction-based only	KT-EL
Coordinate-based only	EL-HT, TR-CL, HT-TR, HT-TS
Both	TS-EL
No similarities	SR, rKT, L1C, RS, TL

To perform the IGNB analysis, different interaction-based structural features such as interaction-based alignment score, alignment length, alignment RMSD, number of matching base pairs, and number of base stackings are generated using the tool RNAmotifScanX (Zhong and Zhang 2015). First, we determined a representative motif for the selected motif families EL, TS, KT, and SR. For each motif family, an instance that best aligns with all other instances is selected as the representative motif. Having the highest alignment score with a lowest RSMD value is considered as a best alignment. In the next step, we calculated the interaction-based 3D structural features for each motif with respect to all four representative motifs. For CGNB analysis, coordinate-based 3D structure alignment features such as alignment length, RMSD, and TM-score have been generated using the tool RNA-align (Gong et al. 2019). A similar approach to the previous analysis is followed where each motif is aligned against the representative motifs from the five selected families EL, TS, HT, CL, and SR to generate their coordinate-based features.

For each binary classifier, the motifs belong to one of the two classes, Class 1 and Class 0, where Class 1 represents the motifs belonging to a certain family and Class 0 represents the motifs that do not belong to a certain family. Then using the random undersampling technique, an equal number of motifs were selected for both classes. For example, in the IGNB analysis, to assess the performance of the E-loop GNB binary classifier, the first 235 motifs belonging to the selected four families (E-loop, Tandem-shear, and Kink-turn, Sarcin-ricin) are taken. From there, the 49 motifs belonging to the E-loop family are assigned to Class E-loop (Class 1) and the remaining motifs are assigned to Class non-E-loop (Class 0). Then a random undersampling is performed so that both Class E-loop and non-E-loop contain an equal number of motifs (49 motifs). A similar approach was followed for all the GNB binary classifiers to handle data bias. Then, to evaluate the performance of each binary classifier, repeated stratified 3-fold cross-validation is used to split the dataset into three non-overlapping folds, maintaining the same class ratio throughout the three folds as the ratio in the original dataset. At each run, one fold is used as the testing dataset while the other two folds are combined and used as the training dataset. After training the GNB model using the training dataset, the performance of the model for that run is tested using the non-overlapping testing dataset. For each model, there were a total of 15 runs (3 splits * 5 repeats), and performance is evaluated by considering the average of accuracy, sensitivity, and specificity of these 15 runs. Following this approach, for both IGNB and CGNB analysis, mean accuracy, specificity, and sensitivity have been calculated. Here, accuracy represents the percentage of correctly identified labels, sensitivity defines the percentage of correctly identified positive labels (Class 1), and specificity defines the percentage of correctly identified negative labels (Class 0).

## 3 Results

By analyzing the internal loop motifs from the input dataset, RNAMotifComp generates a graph that represents the similarity relationship among the motif families. The image generated from our visualization method depicts their relation with associated data. Then we verified our experimental result using RNAMotifContrast (Islam et al. 2021) which mostly supports our findings. Additionally, we have employed a basic Naïve Bayes classifier to demonstrate the utilities of our method. This highlights how the performance of a machine learning model is affected due to the structural similarity among the motif instances from different families. The result from our analysis and the outcome of using RNAMotifContrast as well as the utility of the analysis based on a machine learning model is discussed in the following subsections.

### 3.1 Similarities among the known motif families

**Figure 3.**
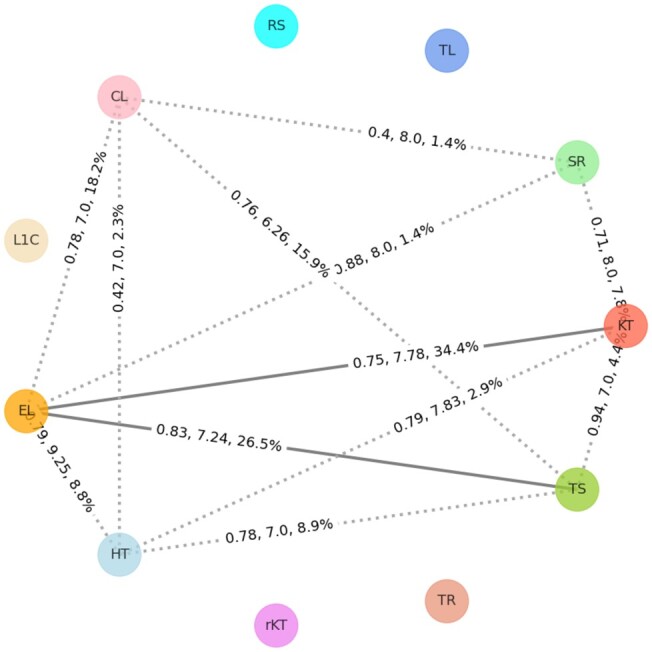
Motif family similarity graph based on base interactions. The edges are calculated by following the procedure described in the “Materials and methods” section. The solid lines represent similar motif families while the dotted lines represent nearly similar families.

**Figure 4.**
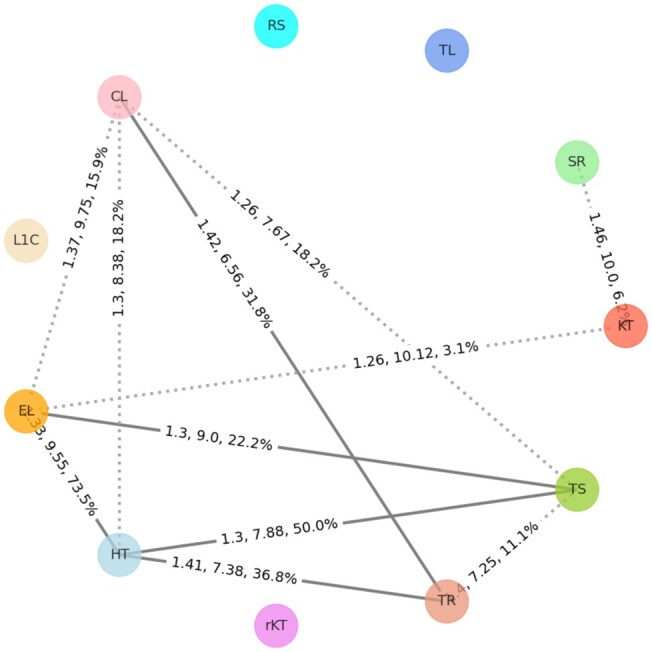
Motif family similarity graph based on the 3D coordinates. The edges are calculated by following the same procedure described in the “Materials and methods” section. The solid and dotted lines represent the similar and nearly similar motif families, respectively.

The generated similarity graph based on the interaction-based and the coordinate-based resemblance is shown in Figs 3 and 4, respectively. A brief summary of the relationship among the motif families found in the two figures is provided in Table 2. In the similarity graphs, we added an edge between two motif families if the average RMSD value of all best-aligned pairs is less than the threshold value. The first two numbers in the edge label represent the average RMSD and average alignment length of selected alignment pairs. The percentage in the edge label represents the proportion of instances of each motif family that are participating in the selected alignments with respect to the total instances in that family. The criteria to find the best-aligned pairs, calculate the average RMSD, average alignment length, and the percentage of participating motif instances along with specific threshold values are briefly described in the “Compute similarities between two motif families” subsection under the “Materials and methods” section. In both Figs 3 and 4, the similarity between the two families is represented using solid lines, and a 20% threshold for the participating motif instances is used to depict similar motif families in the figures. The nearly similar motif families having ˂20% similar instances are shown using dotted lines. This 20% cutoff is selected by manually analyzing the resulting similar motif families for different cutoff values. Additionally, detailed similarity information for each edge is provided in Supplementary Tables S3 and S4 for interactions-based and coordinate-based similarity, respectively.

According to the results shown in Figs 3 and 4, and as summarized in Table 2, E-loop and Tandem-shear motifs are structurally similar from both interaction and coordinate points of view. Based on the selected alignment pairs, the average RMSD between these two motif families are 0.83 Å and 1.30 Å while using interaction-based and coordinate-based alignments, respectively. Moreover, at least 26.5% of instances of both families in Fig. 3 and 22.2% of instances in Fig. 4 are close to each other between E-loop and Tandem-shear motif instances. In exact numbers, 13 E-loop instances are close to 25 Tandem-shear instances based on their interactions while 31 E-loop instances are close to 10 Tandem-shear instances based on their 3D coordinates.

**Figure 5.**
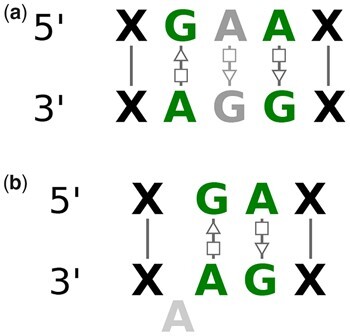
The consensus secondary structures of (a) E-loop motif family based on 13 motifs that were similar to Tandem-shear motifs, and (b) Tandem-shear motif family based on 25 motifs that were similar to E-loop motifs. The proposed notations in the work by Leontis et al. (2002) are used to represent the non-canonical base pairings in this figure.

To get a clear idea, we have generated the consensus structure of the interaction-based similar E-loop and Tandem-shear instances given in Fig. 5. The figure shows that the motifs belonging to E-loop and Tandem-shear families have common tandem G-A/A-G Trans Hoogsteen/Sugar edge interactions. It is important to note that the consensus structure shown here is only based on similar instances. For the consensus structure based on all the instances, please refer to Supplementary Fig. S1. Although the E-loop consensus structure based on all instances shows a Trans Hoogsteen/Watson-Crick base-pairing in the middle of two G-A/A-G Trans Hoogsteen/Sugar pair, the consensus structure of the participating similar instances lacks that pair and instead it has a Trans Hoogsteen/Sugar pair at the same place. We also have generated side-by-side and superimposition images of coordinate-based similar instances from E-loop and Tandem-shear motif families to visualize their structural features. The superimposition image is provided in Fig. 7 while the side-by-side image is given in Supplementary Fig. S2.

**Figure 6.**
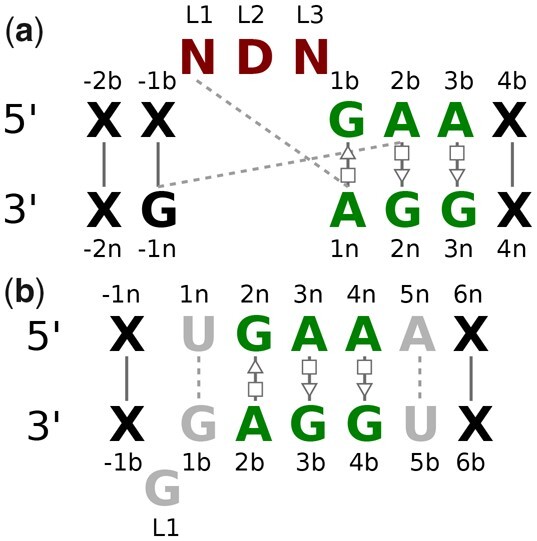
The consensus secondary structures of (a) Kink-turn motif family based on 22 motifs that were similar to E-loop motifs, and (b) E-loop motif family based on 22 motifs that were similar to Kink-turn motifs. The base-pairing notations are used from the same source as Fig. 5, additionally, the nucleotide numbering system is used from the work by Liu and Lilley (2007). In both images, the X represents the terminal nucleotide, the solid lines connecting them represent canonical interaction, and the gray-colored nucleotides including their connecting dashed lines represent less frequently occurring nucleotides and base-pairing interactions, respectively.

Kink-turn and E-loop are both well-known 2-loop motif families (Apostolico et al. 2009). Although there is a huge difference in the 3D structure of these motif families, we found 22 instances from each family that actually share a significant part of their base interactions. We thoroughly checked the interactions of all the participating instances and generated a consensus structure for both families. The consensus structure of Kink-turn and E-loop motif instances are shown in Fig. 6a, b, respectively. As highlighted in this image, 22 motifs from each of the Kink-turn and E-loop families have three common A-G Trans Hoogsteen/Sugar edge interactions. The consensus secondary structures for all the instances in the E-loop and Kink-turn motif family are shown in Supplementary Fig. S1.

**Figure 7.**
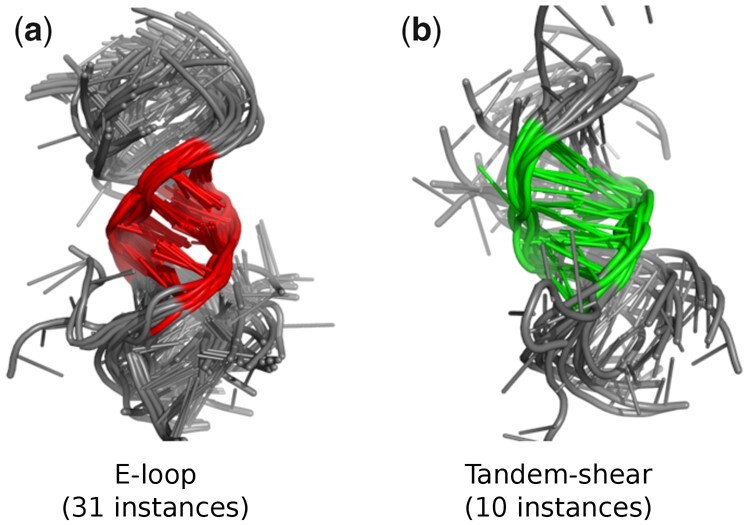
The superimposition image of (a) 31 E-loop motif instances, and (b) 10 Tandem-shear motif instances that are structurally similar from coordinate point of view.

Figure 4 depicts the relationships among the motif families from their coordinate point of view and from that image, the E-loop and Hook-turn pair has the highest similar instances based on their total count. At least 73.5% instances of each motif family are close to the other one or in number, 42 E-loop motifs are close to 25 Hook-turn motif instances from their 3D coordinate point of view. The side-by-side images for these instances are shown in Supplementary Fig. S3. Other than this pair, we also found coordinate-based instance similarities in C-loop/Tetraloop-receptor, Hook-turn/Tetraloop-receptor, and Hook-turn/Tandem-shear motif family pairs.

### 3.2 Comparison with RNAMotifContrast

To see the relationship among the motif instances from different aspects, we have employed RNAMotifContrast (Islam et al. 2021) as a clustering tool on a totally unannotated version of our input instances. A summary of the outcome from both interaction-based and coordinate-based alignment is provided in Table 3. The distinct set of motif families with interaction and/or coordinate-based similarity that have been clustered together has been highlighted with the bold-faced font. The detailed result of both runs is provided in Supplementary Tables S5 and S6, respectively. Both interaction-based 3D structurally similar pairs (EL-KT, EL-TS) and coordinate-based 3D structurally similar pairs (EL-TS, EL-HT, TR-CL, HT-TR, and HT-TS) from our study have been clustered together by RNAmotifContrast as shown in Table 3 which shows consistency in our analysis and RNAmotifContrast clustering output.

**Table 3.** Annotated clusters from RNAMotifContrast result (structurally similar motif families identified by RNAMotifComp are bold-faced).

Clustering criteria	Distinct set of motif families clustered together
Based on interactions	SR, **TS**, **EL**, CL, L1C
	HT, EL, CL
	**KT**, **EL**, CL
	rKT, HT, TS, KT
	KT, SR
	TR, HT
Based on 3D coordinates	**TS**, **EL**, SR, KT, **HT**, CL
	KT, **EL**, rKT, **HT**

One important point to be noted here is, although the RNAMotifContrast result supports our analyzed outcome, it does not fully match our findings. For example, both SR and L1C are assigned to the category of motif families that do not have interaction-based or coordinate-based structural similarity from our analysis which is summarized in Table 2. However, Table 3 shows that RNAMotifContrast clusters these two motif families in the category of having interaction-based similarity. The main reason for this contradiction lies behind the purpose and developing criteria of RNAMotifContrast. This tool is developed to discover and visualize the subfamilies of a given motif family. Although it can subdivide a group of input motif instances into multiple subclusters, it expects the instances of the group to be from a single motif family. The parameters and methods of the pipeline were designed that way. So, RNAmotifContrast doesn’t always generate perfectly precise clusters based on structural similarity, and here comes the necessity of such analysis which is done in our current work.

### 3.3 Utility of RNAMotifComp

Characteristics of different known motif families are generally defined based on the frequent occurrence of sequence patterns, 3D structural patterns, base-pairing, and base-stacking interactions. The existing base-interaction and shape-based 3D structural similarity between motif families can often cause indecision while identifying known motif family instances or discovering new motif families. One such example is explored here using the Naïve Bayes classifier as a machine learning model. These 3D structural similarities are increasing the complexity of the model in identifying motif families based on their 3D structural features, thus degrading the overall performance.

In order to verify if a machine learning model faces difficulty while trying to identify motifs of structurally similar families, we used the Naïve Bayes Classifier (Berrar 2018). Here, we showed that the classifier models perform better while identifying motifs from families that have less structural similarity to other RNA motif families. As discussed in the “Materials and methods” section, we have used two GNB models, IGNB and CGNB, for interaction-based and coordinate-based analysis, respectively. The codes and feature sets used for these two analyses are included in the “Benchmarking using ML” directory in our source repository. The outcome of both IGNB and CGNB classifiers along with their Multiclass GNB (MGNB) classifiers are described in the following paragraphs.

For IGNB analysis, four binary classifier models using the GNB algorithm have been trained separately for the four selected families EL, TS, KT, and SR. Each binary classifier predicts if a given motif belongs to a certain motif family or not. For example, the binary classifier for EL is trained based on the data of the E-loop motif family (labeled as Class 1) and the other three motif families (labeled as Class 0). This binary classifier specifically predicts if a given motif family belongs to the E-loop family or not. Finally, to observe the overall performance, an MGNB classifier is used which predicts if a given motif instance belongs to EL, TS, KT, or SR. The performance of the IGNB classifier and the related MGNB classifier is listed in Table 4.

**Table 4.** Prediction accuracy comparison of specific motif families using two different GNB binary classifiers (IGNB and CGNB).

3D structural similarity criteria	Family	Families with similarity	Performance of GNB classifiers			Accuracy of MGNB classifier
			Sensitivity	Specificity	Accuracy	
Based on interaction (IGNB)	EL	TS, KT	77.5%	46.3%	61.9%	80.1%
	TS	EL	89.0%	90.5%	89.8%	
	KT	EL	84.8%	70.6%	77.7%	
	SR	–	98.6%	89.9%	94.3%	

Based on coordinate (CGNB)	EL	HT, TS	87.9%	66.2%	77.1%	73.8%
	TS	HT, EL	88.7%	78.8%	83.7%	
	HT	TS, EL, TR	80.6%	67.3%	73.9%	
	CL	TR	81.8%	81.8%	81.9%	
	SR	–	85.3%	96.7%	91.0%	

Similarly, for CGNB analysis, five separate binary GNB classifiers were built for the five motif families EL, TS, HT, CL, and SR. Then their accuracy along with the sensitivity and specificity are calculated. Finally, an MGNB classifier model is developed to predict if an input motif instance belongs to EL, TS, HT, CL, or SR. The performance of the coordinate-based binary GNB classifiers (CGNB) and the multiclass GNB classifier model (MGNB) are also depicted in Table 4.

**Figure 8.**
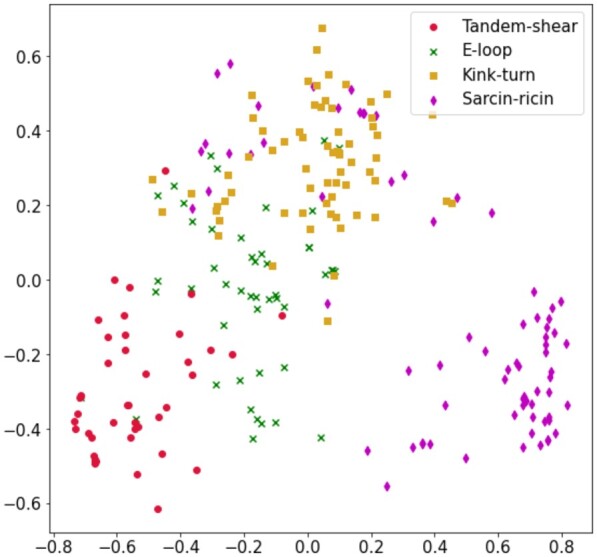
Distribution of Tandem-shear, E-loop, Kink-turn, and Sarcin-ricin motif families formulated from interaction-based 3D structural similarity using PCA technique.

**Figure 9.**
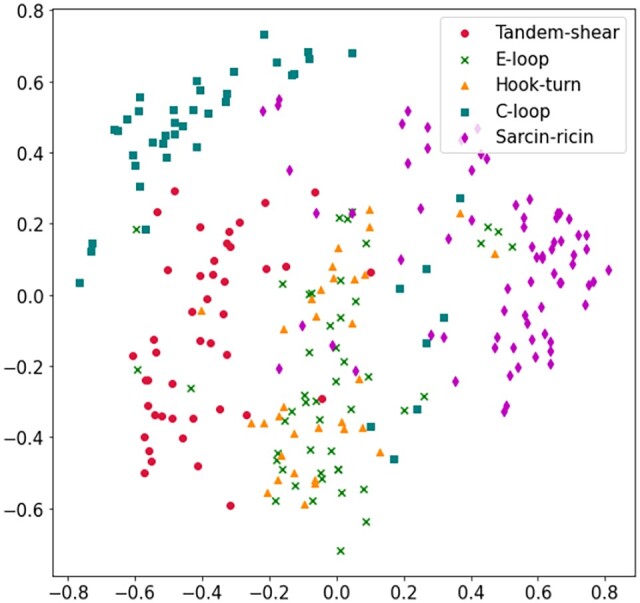
Distribution of Tandem-shear, E-loop, Hook-turn, C-loop, and Sarcin-ricin motif families formulated from coordinate-based 3D structural similarity using PCA technique.

From Table 4, for both interaction and coordinate-based 3D structural similarity, the accuracy for the Sarcin-ricin motif family is the highest (94.3% in IGNB and 91% in CGNB analysis) and the accuracy of the other motif families having structural similarity stays in the lower range compared to Sarcin-ricin. Also, in the case of sensitivity and specificity, Sarcin-ricin seems to score 85% or higher value in both analyses. Overall, the results indicate that, for highly similar motif families, the GNB model faces comparatively higher indecision during prediction which is caused by 3D structural similarities within the families, and thus hampers the prediction accuracy. The result of the GNB models completely aligns with our analysis as on one hand, it is able to identify Sarcin-ricin motifs with higher accuracy which have very low 3D structural similarity, and on the other hand, families having interaction and/or coordinate-based 3D structural similarity have comparatively lower prediction accuracy. The overall accuracy of the interaction-based and coordinate-based MGNB models are 80.1% and 73.8%, respectively, which is comparatively on the lower side of the good accuracy range. Figures 8 and 9 show the distribution of the motif families on a 2D plane based on interaction and coordinate-based structural similarity features, respectively, using the principal component analysis (PCA) technique (Tharwat 2016). Both figures add further proof to our analysis as it can be clearly seen that for both interaction and coordinate-based features, Sarcin-ricin motifs are clustered distinctly while the clusters of the motif families having 3D structural similarity have a higher tendency to overlap.

## 4 Discussion

Besides structural variation among the motif instances of a known motif family, there are also structural similarities within motifs of different motif families. While trying to identify and predict motifs belonging to known motif families, these structural variations and similarities definitely create additional complexity. Due to lack of higher resolution data and annotation errors, annotating the structural motifs can be very confusing for computational algorithms, even for humans. For example, Djelloul and Denise (2008) identified five instances of Tandem-shear motifs, and the authors have mentioned, out of these instances two might be E-loop motifs. Our contribution is to identify structurally close motif instances between two motif families, so that they can be studied more precisely, and re-annotate them if required. These identified similar motif instances across families can also be utilized to pinpoint the base interaction annotation errors. Although we have analyzed only the known internal loop motif families, RNAMotifComp can similarly be used to analyze both hairpin and/or multi loop motif families as both RNAMotifScanX and RNA-align are capable of handling hairpin and multi loops by default. Moreover, users can easily control the RMSD threshold and percentage of participating motif instances threshold through the command line parameters. As RNAMotifComp can identify structurally similar instances from any two sets of motifs, it can be used with RNAMotifContrast or any other motif clustering tool to provide improved clustering results for similar structural motif instances. According to our analysis, E-loop and Tandem-shear families have motif members with similar base interactions and tertiary shapes. Other motif families such as Kink-turn and E-loop have high interaction-based similarity while motifs belonging to families E-loop/Hook-turn/Tandem-shear or Tetraloop-receptor/C-loop or Tetraloop-receptor/Hook-turn contain motifs with coordinate-based similarity. Using RNAMotifContrast and the machine learning prediction method, we have shown how these similarities can create perplexity in motif family identification. So, in future studies, special attention should be paid to these structurally similar families while trying to segregate motifs or assigning newly discovered motifs to any known motif families.

The similarity we discovered from interaction-based alignment could be attempted to be found using a subgraph matching algorithm. But that will require generating a graph representation of each motif instance incorporating all base-pairing and base-stacking interactions of a motif. The complexity will then be no less than the complexity of RNAMotifScanX alignment as it uses maximal clique finding to align one motif instance to another. Moreover, that graph matching algorithm will not work for finding similarities based on the 3D coordinates that we have achieved using the RNAalign tool. Reinharz et al. (2018) and Soulé et al. (2021) have proposed a method to find RNA modules based on recurrent interaction networks (RINs) and interaction modules. Their main focus was on the base-pairing interactions rather than motif regions. Besides, these works rely on the 2D structure graphs which will not be able to identify the structural similarity based on their 3D coordinates. Instead of focusing only on interactions, we followed the same procedure to show the 3D structural similarities from both interaction and coordinate points of view. The analysis we have described in this manuscript and the result we found can be significantly helpful in identifying motif functionality, protein interaction, or RNA evaluation and play a significant role in the structural similarity within certain motif families. This can guide toward designing more sophisticated algorithms to define each motif family that can lead the way toward well-refined motif clusterization and discovery.

## Supplementary Material

btad223_Supplementary_Data

## Data Availability

The data underlying this article are available in GitHub repository at https://github.com/ucfcbb/RNAMotifFamilySimilarity.
