# Supplementary material for: RNAMotifComp: a comprehensive method to analyze and identify structurally similar RNA motif families
Source: Bioinformatics. 2023 Jun 30;39(Suppl 1):i337–46. doi: 10.1093/bioinformatics/btad223 (PMC10311341; doi:10.1093/bioinformatics/btad223)
Supplement: btad223_Supplementary_Data [file btad223_supplementary_data.pdf]

# Supplementary Materials for "RNAMotifComp: a comprehensive method to analyze and identify structurally similar RNA motif families"

Md Mahfuzur Rahaman, Nabila Shahnaz Khan, Shaojie Zhang*

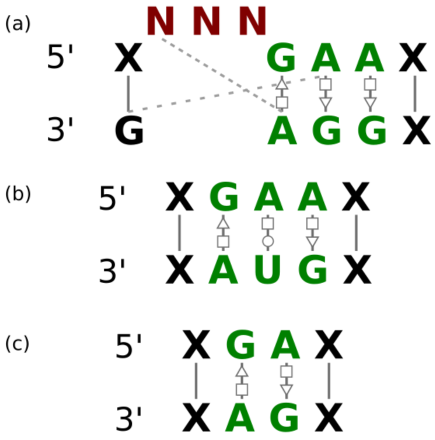


Figure S1: The consensus secondary structures with non-canonical interactions based on all the motif instances of Kink-turn, E-loop and Tandem-shear motif families. (a) Kink-turn motif family based on 67 motifs (b) E-loop motif family based on 49 motifs (c) Tandem-shear motif family based on 45 motifs. The base-pairing notations as well as the formatting are used from the same source as Figure 5.

*To whom correspondence should be addressed. Tel: +1 407 8236095; Fax: +1 407 8235835; Email: shzhang@cs.ucf.edu

Table S1: Intra and inter-family RMSD distribution for interaction-based alignment

| | Interaction-based alignment | | | | | |
|---|---|---|---|---|---|---|
| | Intra-family RMSD dist. | | | Inter-family RMSD dist. | | |
| | **<=1.0 Å** | <=1.5 Å | <=2.0 Å | **<=1.0 Å** | <=1.5 Å | <=2.0 Å |
| **Kink-turn** | 33.15% | 55.27% | 63.77% | 9.51% | 21.70% | 30.34% |
| **reverse-Kink-turn** | 50.00% | 62.50% | 78.12% | 12.80% | 16.19% | 22.78% |
| **Sarcin-ricin** | 60.56% | 67.03% | 72.31% | 13.71% | 23.50% | 29.00% |
| **C-loop** | 24.75% | 38.12% | 57.00% | 16.10% | 28.14% | 36.36% |
| **E-loop** | 37.17% | 59.44% | 74.11% | 14.92% | 30.64% | 40.71% |
| **Hook-turn** | 46.71% | 61.94% | 82.01% | 12.15% | 23.15% | 30.41% |
| **Tandem-shear** | 58.88% | 71.49% | 80.99% | 19.96% | 31.64% | 40.51% |
| **Tetraloop-receptor** | 80.62% | 88.93% | 90.31% | 16.35% | 25.20% | 36.57% |
| **L1-complex** | 61.11% | 77.78% | 77.78% | 31.99% | 46.06% | 54.09% |
| **Rope-sling** | 95.92% | 100.00% | 100.00% | 14.11% | 19.22% | 24.32% |
| **T-loop** | 100.00% | 100.00% | 100.00% | 9.76% | 19.23% | 24.41% |
| **Average percentage** | **58.99%** | **71.14%** | **79.67%** | **15.58%** | **25.88%** | **33.59%** |

Table S2: Intra and inter-family RMSD distribution for coordinate-based alignment

| | Coordinate-based alignment | | | | | |
|---|---|---|---|---|---|---|
| | Intra-family RMSD dist. | | | Inter-family RMSD dist. | | |
| | <=1.0 Å | **<=1.5 Å** | <=2.0 Å | <=1.0 Å | **<=1.5 Å** | <=2.0 Å |
| **Kink-turn** | 13.33% | 21.97% | 39.55% | 4.74% | 19.22% | 42.71% |
| **reverse-Kink-turn** | 34.38% | 37.50% | 90.62% | 10.95% | 31.33% | 48.56% |
| **Sarcin-ricin** | 32.99% | 43.27% | 49.73% | 5.89% | 18.85% | 38.50% |
| **C-loop** | 20.75% | 40.75% | 56.75% | 9.72% | 30.91% | 53.66% |
| **E-loop** | 17.52% | 39.43% | 75.19% | 7.41% | 22.52% | 49.70% |
| **Hook-turn** | 33.04% | 50.35% | 78.55% | 6.78% | 18.52% | 43.11% |
| **Tandem-shear** | 49.48% | 79.96% | 89.57% | 8.46% | 30.81% | 61.69% |
| **Tetraloop-receptor** | 39.79% | 52.94% | 56.40% | 9.86% | 29.81% | 56.92% |
| **L1-complex** | 72.22% | 94.44% | 100.00% | 3.41% | 15.11% | 32.33% |
| **Rope-sling** | 91.84% | 100.00% | 100.00% | 15.80% | 36.04% | 57.92% |
| **T-loop** | 50.00% | 100.00% | 100.00% | 5.36% | 14.14% | 27.53% |
| **Average percentage** | **41.39%** | **60.06%** | **76.03%** | **8.03%** | **24.30%** | **46.60%** |

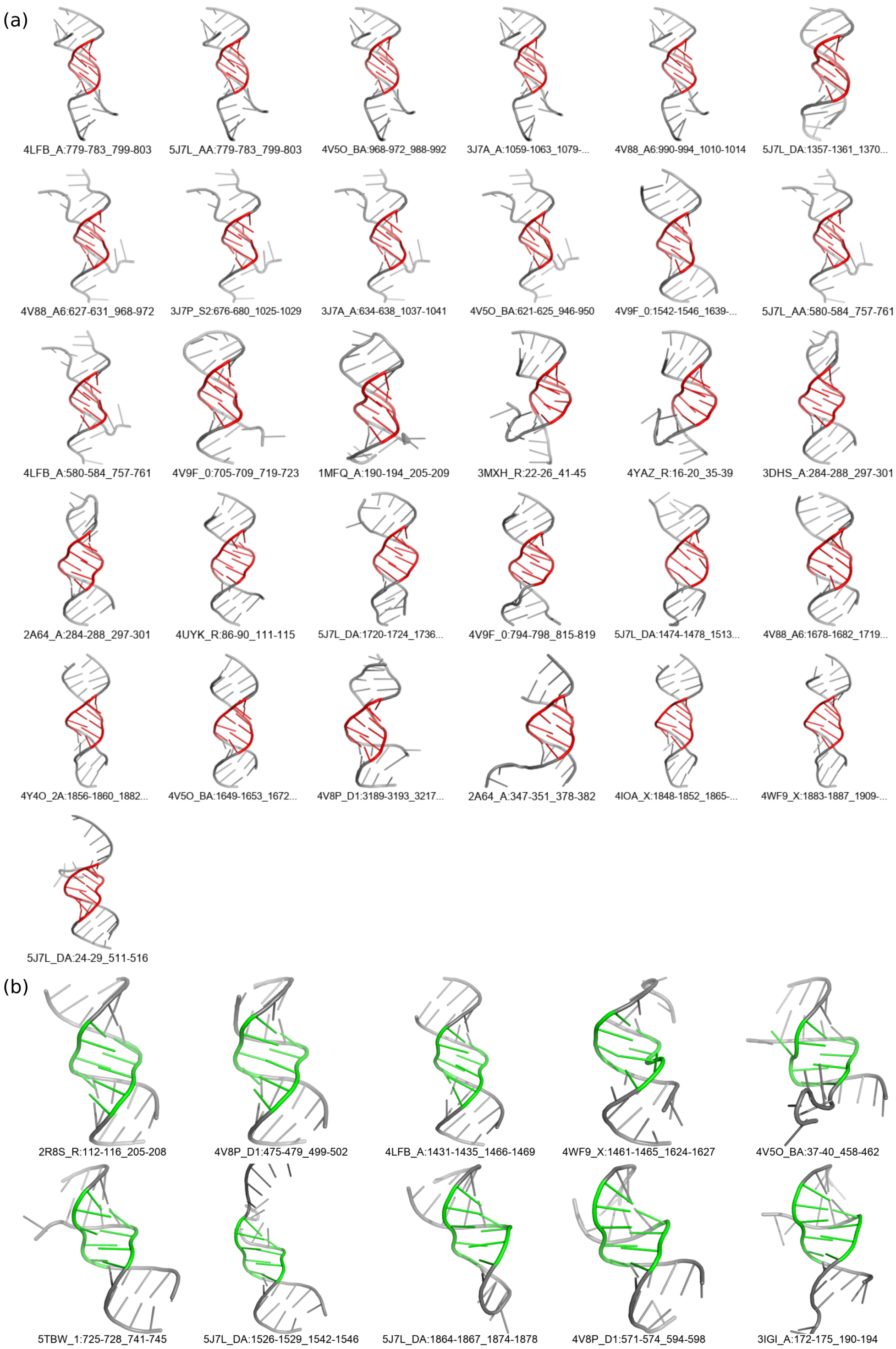


Figure S2: The side-by-side representation of E-loop and Tandem-shear motif instances that are found similar based on our analysis. (a) E-loop motif instances, (b) Tandem-shear motif instances. The similarity is calculated based on the coordinate-based alignments.

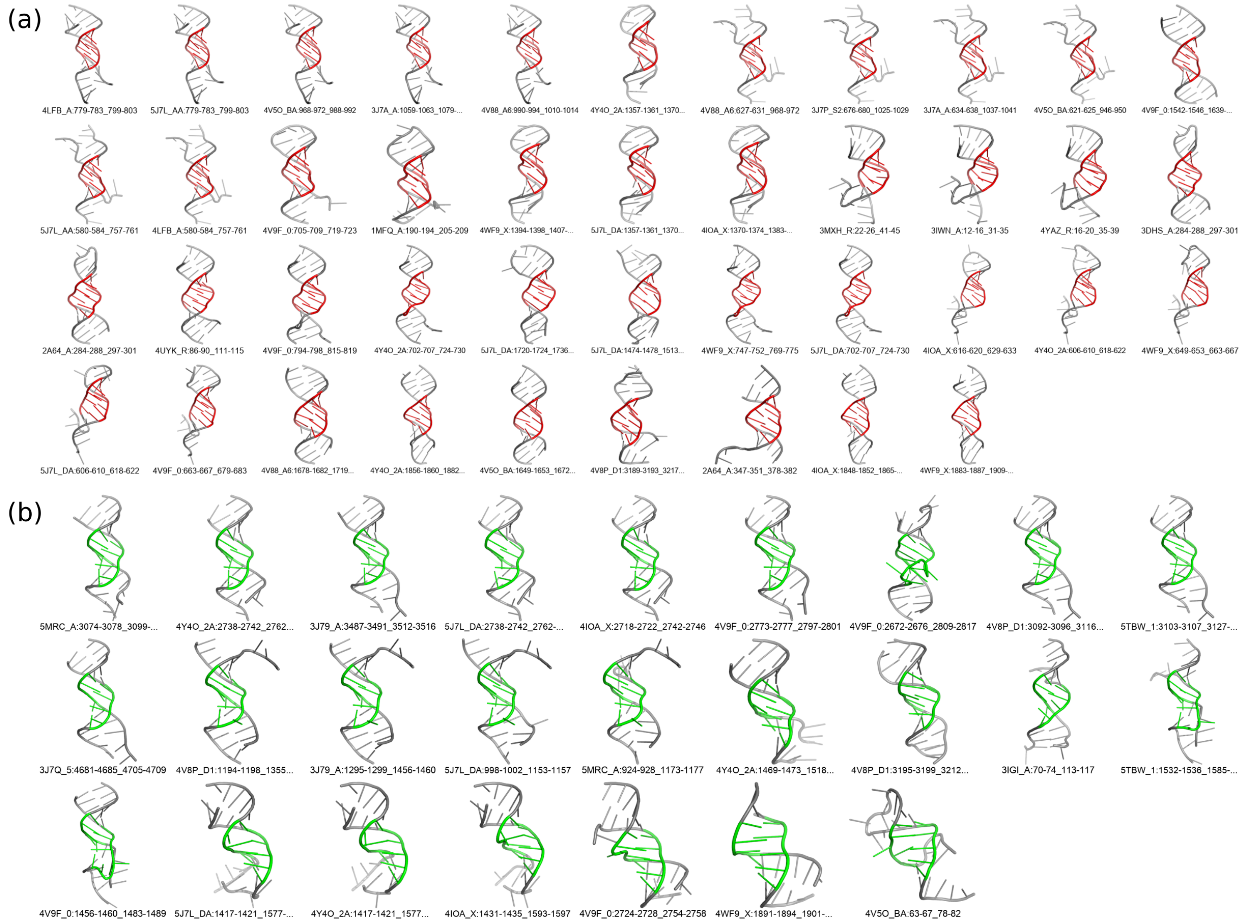


Figure S3: The side-by-side representation of E-loop and Hook-turn motif instances that are found similar based on our analysis. (a) E-loop motif instances, (b) Hook turn motif instances. The similarity is calculated based on the coordinate-based alignments.

Table S3: Similarity information of the input motif families using interaction-based alignment

| **Motif Family1 (MF1)** | **Motif Family2 (MF2)** | **Mean RMSD** | **Mean alignment length** | **Participating instances from MF1** | **Participating instances from MF2** | **Edge label** |
|---|---|---|---|---|---|---|
| KT | SR | 0.71 | 8.0 | 5 / 64 (7.81%) | 15 / 74 (20.27%) | 0.71, 8.0, 7.8% |
| KT | EL | 0.75 | 7.78 | 22 / 64 (34.38%) | 22 / 49 (44.9%) | **0.75, 7.78, 34.4%** |
| KT | HT | 0.79 | 7.83 | 6 / 64 (9.38%) | 1 / 34 (2.94%) | 0.79, 7.83, 2.9% |
| KT | TS | 0.94 | 7.0 | 4 / 64 (6.25%) | 2 / 45 (4.44%) | 0.94, 7.0, 4.4% |
| SR | CL | 0.4 | 8.0 | 1 / 74 (1.35%) | 3 / 44 (6.82%) | 0.4, 8.0, 1.4% |
| SR | EL | 0.88 | 8.0 | 1 / 74 (1.35%) | 2 / 49 (4.08%) | 0.88, 8.0, 1.4% |
| CL | EL | 0.78 | 7.0 | 8 / 44 (18.18%) | 13 / 49 (26.53%) | 0.78, 7.0, 18.2% |
| CL | HT | 0.42 | 7.0 | 1 / 44 (2.27%) | 1 / 34 (2.94%) | 0.42, 7.0, 2.3% |
| CL | TS | 0.76 | 6.26 | 7 / 44 (15.91%) | 17 / 45 (37.78%) | 0.76, 6.26, 15.9% |
| EL | HT | 0.79 | 9.25 | 8 / 49 (16.33%) | 3 / 34 (8.82%) | 0.79, 9.25, 8.8% |
| EL | TS | 0.83 | 7.24 | 13 / 49 (26.53%) | 25 / 45 (55.56%) | **0.83, 7.24, 26.5%** |
| HT | TS | 0.78 | 7.0 | 21 / 34 (61.76%) | 4 / 45 (8.89%) | 0.78, 7.0, 8.9% |

Table S4: Similarity information of the input motif families using coordinate-based alignment

| **Motif Family1 (MF1)** | **Motif Family2 (MF2)** | **Mean RMSD** | **Mean alignment length** | **Participating instances from MF1** | **Participating instances from MF2** | **Edge label** |
|---|---|---|---|---|---|---|
| KT | SR | 1.46 | 10.0 | 4 / 64 (6.25%) | 5 / 74 (6.76%) | 1.46, 10.0, 6.2% |
| KT | EL | 1.26 | 10.12 | 2 / 64 (3.12%) | 7 / 49 (14.29%) | 1.26, 10.12, 3.1% |
| CL | EL | 1.37 | 9.75 | 7 / 44 (15.91%) | 21 / 49 (42.86%) | 1.37, 9.75, 15.9% |
| CL | HT | 1.3 | 8.38 | 8 / 44 (18.18%) | 14 / 34 (41.18%) | 1.3, 8.38, 18.2% |
| CL | TR | 1.42 | 6.56 | 14 / 44 (31.82%) | 14 / 19 (73.68%) | **1.42, 6.56, 31.8%** |
| CL | TS | 1.26 | 7.67 | 8 / 44 (18.18%) | 22 / 45 (48.89%) | 1.26, 7.67, 18.2% |
| EL | HT | 1.33 | 9.55 | 42 / 49 (85.71%) | 25 / 34 (73.53%) | **1.33, 9.55, 73.5%** |
| EL | TS | 1.3 | 9.0 | 31 / 49 (63.27%) | 10 / 45 (22.22%) | **1.3, 9.0, 22.2%** |
| HT | TR | 1.41 | 7.38 | 13 / 34 (38.24%) | 7 / 19 (36.84%) | **1.41, 7.38, 36.8%** |
| HT | TS | 1.3 | 7.88 | 17 / 34 (50.0%) | 43 / 45 (95.56%) | **1.3, 7.88, 50.0%** |
| TR | TS | 1.4 | 7.25 | 4 / 19 (21.05%) | 5 / 45 (11.11%) | 1.4, 7.25, 11.1% |

Table S5: RNAMotifContrast output using RNAMotifScanX alignment with the unannotated data containing all input motif instances

| Motif cluster | # Motif members | # Motifs belonging to known motif families |
|---|---|---|
| IL-Sub1 | 105 | SR (54), TS (35), CL (7), EL (6), L1C (3) |
| IL-Sub2 | 3 | L1C(2), TS(1) |
| IL-Sub3 | 21 | rKT (7), HT (7), TS (4), KT (3) |
| IL-Sub4 | 10 | HT (10) |
| IL-Sub5 | 3 | HT (2), rKT (1) |
| IL-Sub6 | 10 | HT (6), EL (3), CL (1) |
| IL-Sub7 | 2 | KT (2) |
| IL-Sub8 | 3 | EL (2), HT (1) |
| IL-Sub9 | 2 | EL (2) |
| IL-Sub10 | 39 | KT (28), EL (10), CL (1) |
| IL-Sub11 | 7 | EL (7) |
| IL-Sub12 | 3 | TS (2), HT (1) |
| IL-Sub13 | 3 | TS (2), HT (1) |
| IL-Sub14 | 2 | EL (2) |
| IL-Sub15 | 16 | EL (16) |
| IL-Sub16 | 21 | KT (21) |
| IL-Sub17 | 5 | SR (5) |
| IL-Sub18 | 3 | SR (3) |
| IL-Sub19 | 4 | KT (4) |
| IL-Sub20 | 2 | L1C (1), EL (1) |
| IL-Sub21 | 3 | KT (2), SR (1) |
| IL-Sub22 | 4 | HT (4) |
| IL-Sub23 | 7 | SR (7) |
| IL-Sub24 | 6 | CL (6) |
| IL-Sub25 | 3 | CL (3) |
| IL-Sub26 | 10 | CL (10) |
| IL-Sub27 | 6 | CL (6) |
| IL-Sub28 | 2 | CL (2) |
| IL-Sub29 | 15 | TR (14), HT (1) |
| IL-Sub30 | 3 | KT (3) |
| IL-Sub31 | 5 | CL (5) |
| IL-Sub32 | 3 | SR (3) |
| IL-Sub33 | 2 | TR (2) |
| IL-Sub34 | 2 | TL (2) |
| IL-Sub35 | 6 | RS (6) |
| IL-Sub36 | 3 | RS (3) |

Table S6: RNAMotifContrast output using RNA-align alignment with the unannotated data containing all input motif instances

| Motif cluster | # Motif members | # Motifs belonging to known motif families |
|---|---|---|
| IL-Sub1 | 93 | TS(38), EL(27), SR(13), KT(10), HT(4), CL(1) |
| IL-Sub2 | 5 | TS (5) |
| IL-Sub3 | 7 | CL (7) |
| IL-Sub4 | 4 | KT (4) |
| IL-Sub5 | 4 | KT (4) |
| IL-Sub6 | 2 | rKT (2) |
| IL-Sub7 | 24 | KT (12), EL (7), rKT (3), HT(2) |
| IL-Sub8 | 4 | EL (4) |
| IL-Sub9 | 2 | rKT (2) |
| IL-Sub10 | 3 | KT (3) |
| IL-Sub11 | 42 | SR (41), EL (1) |
| IL-Sub12 | 2 | SR (2) |
| IL-Sub13 | 4 | EL (4) |
| IL-Sub14 | 13 | KT (13) |
| IL-Sub15 | 23 | HT (23) |
| IL-Sub16 | 10 | KT (10) |
| IL-Sub17 | 5 | EL (5) |
| IL-Sub18 | 5 | KT (5) |
| IL-Sub19 | 3 | HT (3) |
| IL-Sub20 | 10 | TR (10) |
| IL-Sub21 | 7 | SR (7) |
| IL-Sub22 | 6 | L1C (6) |
| IL-Sub23 | 7 | SR (7) |
| IL-Sub24 | 5 | CL (5) |
| IL-Sub25 | 5 | TR (5) |
| IL-Sub26 | 3 | SR (3) |
| IL-Sub27 | 19 | CL (19) |
| IL-Sub28 | 3 | KT (3) |
| IL-Sub29 | 2 | TL (2) |
| IL-Sub30 | 10 | RS (10) |
| IL-Sub31 | 5 | CL (5) |
| IL-Sub32 | 4 | CL (4) |
| IL-Sub33 | 2 | TR (2) |
| IL-Sub34 | 2 | CL (2) |
